# The Internally Truncated LRP5 Receptor Presents a Therapeutic Target in Breast Cancer

**DOI:** 10.1371/journal.pone.0004243

**Published:** 2009-01-22

**Authors:** Peyman Björklund, Jessica Svedlund, Anna-Karin Olsson, Göran Åkerström, Gunnar Westin

**Affiliations:** 1 Department of Surgical Sciences, Endocrine Unit, Uppsala University, Uppsala University Hospital, Uppsala, Sweden; 2 Department of Medical Biochemistry and Microbiology, Uppsala University, Uppsala Biomedical Center, Uppsala, Sweden; Northwestern University, United States of America

## Abstract

**Background:**

Breast cancer is a common malignant disease, which may be caused by a number of genes deregulated by genomic or epigenomic events. Deregulated WNT/β-catenin signaling with accumulation of β-catenin is common in breast tumors, but mutations in WNT signaling pathway components have been rare. An aberrantly spliced internally truncated LRP5 receptor (LRP5Δ666–809, LRP5Δ) was shown recently to be resistant to DKK1 inhibition, and was required for β-catenin accumulation in hyperparathyroid tumors and parathyroid tumor growth.

**Methodology/Principal Findings:**

Here we show, by reverse transcription PCR and Western blot analysis, that LRP5Δ is frequently expressed in breast tumors of different cancer stage (58–100%), including carcinoma *in situ* and metastatic carcinoma. LRP5Δ was required in MCF7 breast cancer cells for the non-phosphorylated active β-catenin level, transcription activity of β-catenin, cell growth *in vitro*, and breast tumor growth in a xenograft SCID mouse model. WNT3 ligand, but not WNT1 and WNT3A augmented the endogenous β-catenin activity of MCF7 cells in a DKK1-insensitive manner. Furthermore, an anti-LRP5 antibody attenuated β-catenin activity, inhibited cell growth, and induced apoptosis in LRP5Δ-positive MCF7 and T-47D breast cancer cells, but not in control cells.

**Conclusions/Significance:**

Our results suggest that the LRP5Δ receptor is strongly implicated in mammary gland tumorigenesis and that its aberrant expression present an early event during disease progression. LRP5 antibody therapy may have a significant role in the treatment of breast cancer.

## Introduction

Deregulated Wnt signaling with accumulation of β-catenin in the cytoplasm/nucleus plays an important role in a variety of human cancers. The binding of WNT ligand to frizzled and LRP5/6 cell surface receptors normally leads to inhibition of a “destruction complex” consisting of APC/Axin/GSK-3β/Ck1/Dvl and other factors, with subsequent accumulation of dephosphorylated stabilized β-catenin, and regulation of its target genes [1]–[7]. Wnt signaling is involved in mammary gland development and mouse WNT1, WNT3, and WNT10b are clearly implicated in MMTV-induced breast tumorigenesis [8], [9]. The LRP5 receptor was recently shown to be required for mammary ductal stem cell activity and WNT1-induced tumorigenesis in the mouse [10]. This may be of particular importance since subtypes of breast tumors have been suggested to originate from stem cell populations [11]–[13].

Aberrant activation of Wnt signaling by cytoplasmic/nuclear β-catenin has been reported in over 60% of human breast cancers [14]–[19]. Mutation of APC, AXIN or CTNNB1 (β-catenin), frequently observed in colorectal cancer, is however rare in breast cancer [9], [20]. Other mechanisms proposed to be involved include increased expression of WNT ligands or DVL1, and epigenetic inactivation of WNT pathway antagonists SFRPs, DKK1, and WIF1 [13], [21]–[25].

A novel mechanism for aberrant activation of Wnt signaling was recently disclosed by us in parathyroid tumors [26]. An aberrantly spliced internally truncated LRP5 receptor (LRP5Δ666–809, LRP5Δ) was found to be expressed in the majority of analyzed parathyroid tumors of both primary and secondary origin, which all displayed cytoplasmic/nuclear β-catenin. The LRP5Δ receptor responded strongly to WNT3 ligand and was shown to be required for accumulation of nonphosphorylated transcriptionally active β-catenin, MYC expression, parathyroid cell growth *in vitro*, and parathyroid tumor growth *in vivo* in SCID mice. Furthermore, LRP5Δ and stabilizing mutation of *CTNNB1* was found to be mutually exclusive in those tumors [26], [27]. The 142 amino acid (666–809) extracellular truncation of LRP5Δ overlaps a binding domain for the LRP5 antagonist DKK1 [28]–[31], and consequently LRP5Δ was found to be insensitive to inhibition by the DKK1 ligand [26].

We have now extended our analysis of aberrant Wnt signaling to breast carcinoma, and demonstrate a fundamental role for the internally truncated LRP5 receptor in deregulated β-catenin signaling and tumor growth.

## Results and Discussion

### The LRP5Δ receptor is expressed in breast carcinoma

**Figure 1:**
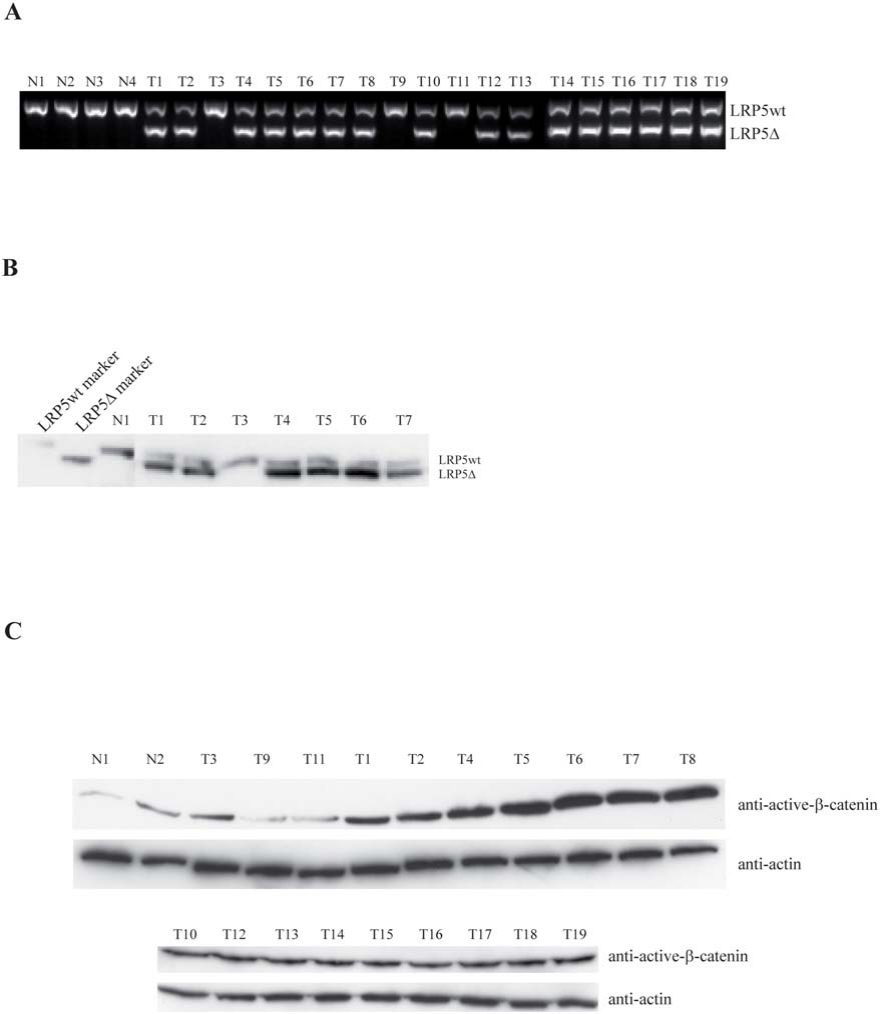
The LRP5Δ receptor is expressed in breast tumors. Accumulation of active β-catenin. (A) PCR analysis of cDNA from normal breast tissue (N1–N4) and primary breast cancer (T1–T19). Primers were located in exons 9 and 13 of LRP5 as described [26]. The PCR reaction is not quantitative as the primers compete for the two fragments. A total of sixty-two LRP5 truncated fragments (see also Table 1) were directly sequenced and all contained the same in-frame deletion of 142 amino acids (Δ666–809). (B) Immunoprecipitation and Western blot analysis of LRP5. Tissue numbering corresponds to panel A. Transiently expressed LRP5wt and LRP5Δ shown as markers. (C) Western blot analysis of non-phosphorylated active β-catenin. Tissue numbering as in panel A.

To investigate whether the internally truncated LRP5Δ666–809 receptor (LRP5Δ) was expressed in breast carcinoma we initially analyzed nineteen breast cancer specimens (T1–T19) that had been randomly selected in a previous study regarding 25-hydroxyvitamin D_3_ 1α-hydroxylase [32]. RT-PCR analysis, using primers located in exons 9 and 13 of LRP5 [26], revealed expression of LRP5Δ in 15 out of 17 ductal breast carcinoma and 1 out of 2 lobular carcinoma (Figure 1A). Thus, 84% of the analyzed tumors expressed LRP5Δ. Normal LRP5 (LRP5wt) transcripts were seen in all tumors, and in normal breast tissue specimens (N1–N4) as anticipated (Figure 1A) [26]. Immunoprecipitation and Western blot analysis confirmed expression of LRP5wt and LRP5Δ in the tumors (Figure 1B).

In order to relate accumulation of β-catenin to expression of LRP5Δ, Western blotting analysis was done on cryosections of the tumor material, including two normal breast tissue specimens. The sixteen tumors with LRP5Δ showed increased accumulation of non-phosphorylated active β-catenin when compared to the normal breast tissues, and the tumors expressing LRP5wt only (T3, T9, T11) showed similar relative amounts (Figure 1C). Thus, correlation of LRP5Δ expression and aberrant accumulation of non-phosphorylated active β-catenin was observed in these breast tumors. Stabilizing mutation in β-catenin exon 3 was not found in the nineteen breast carcinomas (not shown).

Encouraged by the above results we then screened a commercially available cDNA panel of 96 breast tissue specimens that covered eight cancer stages. LRP5wt and LRP5Δ was observed in 79 out of 95 (83%) samples (Table 1). The remaining samples expressed only LRP5wt and one sample was negative. LRP5Δ was detected in all disease stages including carcinoma *in situ* and metastases. Thus, expression of LRP5Δ was very common in carcinoma of the breast and may be an early event during cancer progression.

**Table 1:** Expression of LRP5Δ in breast tumor specimens of different cancer stages.

Stage	LRP5Δ	%
0	7 (n = 12)	58%
I	17 (n = 20)	85%
IIA	17 (n = 21)	81%
IIB	10 (n = 13)	77%
IIIA	16 (n = 16)	100%
IIIB	2 (n = 2)	100%
IIIC	6 (n = 7)	86%
IV	4 (n = 4)	100%
Total	79 (n = 95)	83%

### LRP5Δ causes active β-catenin signaling in MCF7 cells

**Figure 2:**
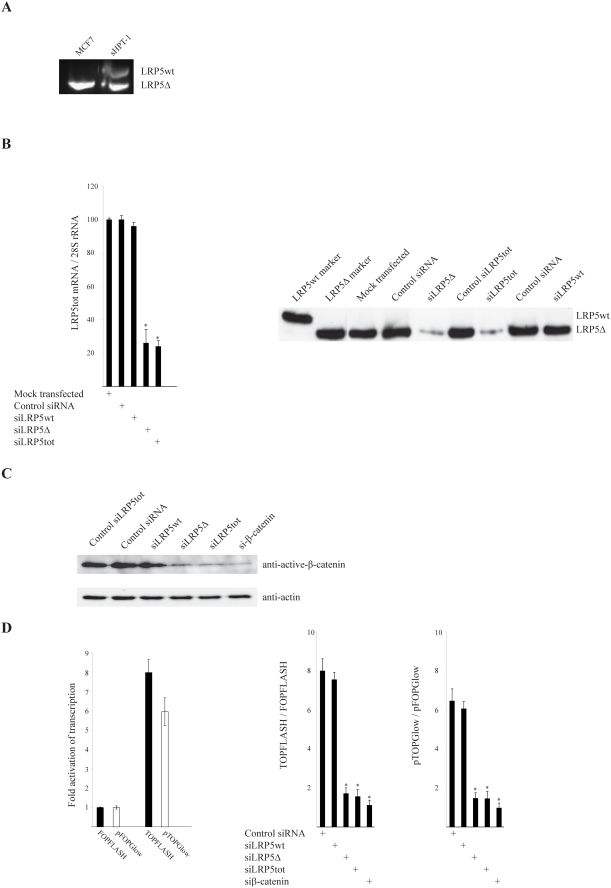
Endogenous expression of LRP5Δ in MCF7 breast cancer cells is required for accumulation of non-phosphorylated active β-catenin and transcriptional activation by β-catenin. (A) PCR of MCF7 cDNA as in panel A. sHPT-1 parathyroid cDNA [26] was used as marker. Direct sequencing confirmed the in-frame deletion of 142 amino acids (Δ666–809). (B) Specificity and efficiency of siRNAs transiently transfected to MCF7 cells. siLRP5wt is directed to wild type transcripts, siLRP5Δ to the truncated transcript, and siLRP5tot to both transcripts [26]. Quantitative real-time PCR of both LRP5 transcripts (LRP5tot, left panel) and immunoprecipitation and Western blot analysis of LRP5 (right panel). (C) Western blot analysis of non-phosphorylated active β-catenin after siRNA transfection. (D) Transient cotransfections of TOPFLASH/FOPFLASH or pTOPGlow/pFOPGlow TCF/β-catenin reporter, the CMV-LacZ reference plasmid (left panel), and the indicated siRNAs (right panels) to MCF7 cells. FOPFLASH and pFOPGlow contain mutated binding sites for TCFs, while TOPFLASH and pTOPGlow do not. Luciferase activities were normalized to β-galactosidase activities. The siRNAs displayed no effect on pFOPGlow (not shown) and FOPFLASH reporter activity [26].

In order to study the consequences of LRP5Δ receptor expression we employed the widely used MCF7 mammary adenocarcinoma cell line which expressed the LRP5Δ receptor but not LRP5wt (Figure 2A), and has been shown to accumulate β-catenin in the nucleus [25], [33]. We first chose to test the TOPFLASH/FOPFLASH and the pTOPGlow/pFOPGlow TCF luciferase reporters, although conflicting results regarding detection of active β-catenin signaling using these reporters in breast cancer cell lines have been published [14], [34], [35].

Control siRNAs and three highly specific siRNAs directed against LRP5 mRNA were transfected to MCF7 cells. The specificity and silencing potential of the LRP5 siRNAs were ascertained at the mRNA and protein level in MCF7 cells (Figure 2B), as we have shown previously in sHPT-1 parathyroid tumor cells [26]. Transfection of siLRP5Δ, specific for the internally truncated LRP5 receptor, as well as of siLRP5tot directed against exon 13 present in both LRP5 wt and LRP5Δ transcripts, resulted in markedly reduced non-phosphorylated active β-catenin level, compared to control siRNAs and siLRP5wt (Figure 2C). siLRP5wt was directed to exon 10, not included in the LRP5Δ transcript. siβ-catenin was included as positive control. Similarily, the endogenous β-catenin activity in MCF7 (Figure 2D, left panel), as measured by using the TOPFLASH/FOPFLASH or the pTOP/Glow/pFOPGlow TCF/β-catenin luciferase reporters (6–8 fold), was dependent on maintained expression of LRP5Δ and β-catenin (Figure 2D, right panels). The various siRNAs displayed no effect on FOPFLASH [26] or pFOPGlow reporter activities (data not shown), which contain mutated TCF binding elements in their promoters [34]. The experiments were done using our in house strain of MCF7 or fresh MCF7 cells from ATCC, which similarily expressed LRP5Δ and showed β-catenin activity by the TOPFLASH and pTOPGlow assays.

**Figure 3:**
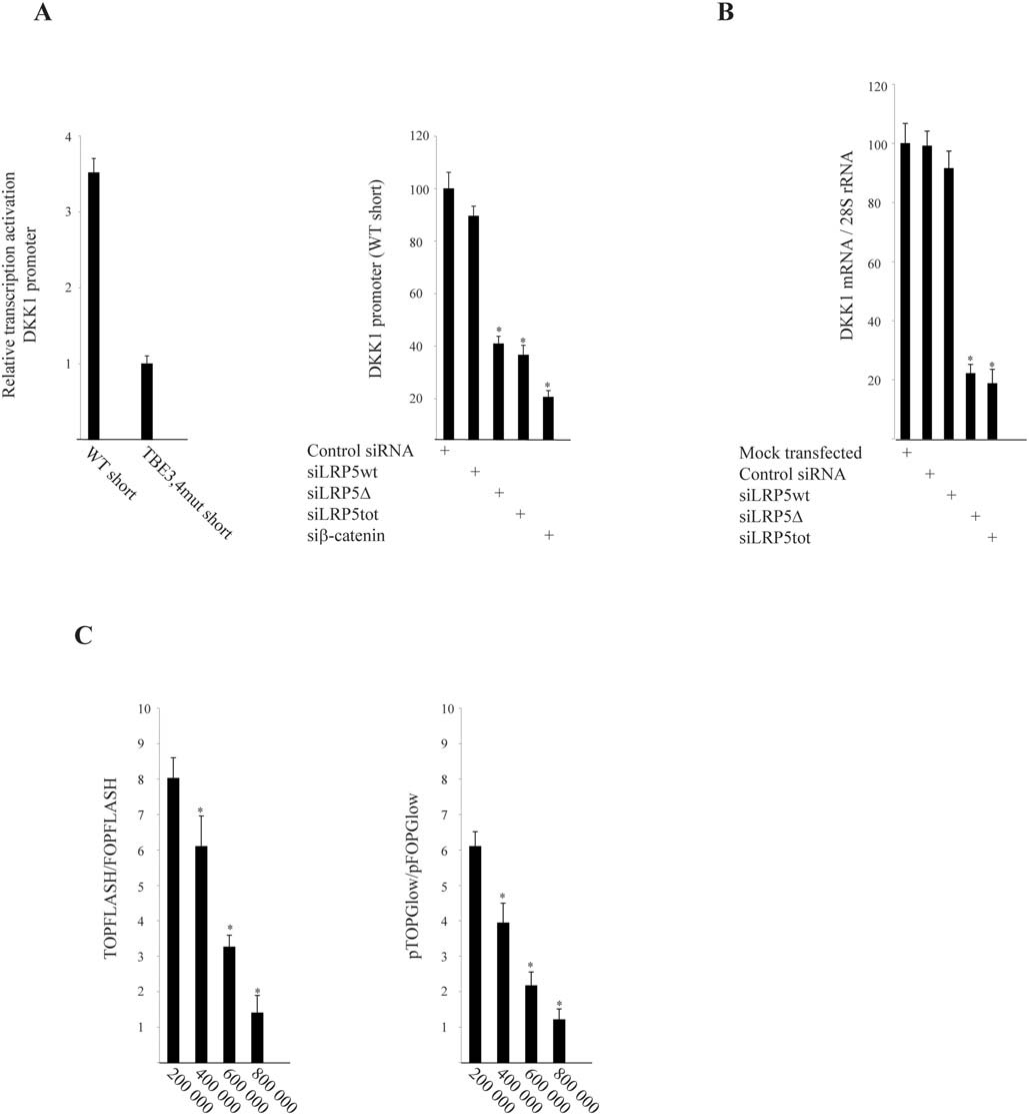
Endogenous expression of LRP5Δ in MCF7 breast cancer cells is required for transcriptional activation of DKK1 by β-catenin. (A) Transient cotransfections of DKK1 promoter luciferase constructs WT short or TBE3,4mut short [38] and CMV-LacZ to MCF7 cells (left panel). WT short, CMV-LacZ, and the indicated siRNAs were transfected (right panel). (B) Endogenous DKK1 mRNA expression in siRNA transfected MCF7 cells as indicated. (C) Transient cotransfections of TOPFLASH/FOPFLASH (left panel) or pTOPGlow/pFOPGlow (right panel) and CMV-LacZ to the indicated number of plated MCF7 cells.

Next we employed the natural β-catenin responsive DKK1 promoter [36]–[38] instead of the synthetic minimal promoters of TOPFLASH and pTOPGlow. Clearly, the DKK1 promoter activity in transfected MCF7 cells was dependent on the TCF binding elements (TBEs), and also on maintained expression of LRP5Δ and β-catenin (Figure 3A). Thus, this confirmed the results obtained with the TOPFLASH and pTOPGlow reporters (Figure 2D). Furthermore, we transfected the various siRNAs to MCF7 cells and determined the endogenous DKK1 mRNA expression level. In accordance with the above results, transfection of siLRP5Δ and siLRP5tot, but not of siLRP5wt or Control siRNA resulted in significantly reduced endogenous DKK1 expression (Figure 3B).

In an attempt to address possible explanations for conflicting results regarding detection of β-catenin activity by the TOPFLASH and pTOPGlow assays [14], [34], [35], we performed transfections in the presence of varying cell densities. We routinely plate cells rather sparsely (2×10^5^ cells/35 mm dish, 10^4^ cells/96-well microplates) as compared to for example 80% confluency [34]. As expected, the transfection efficiency (β-galactosidase activity) was reduced with increasing cell density (not shown), and clearly the TOPFLASH/FOPFLASH as well as the pTOPGlow/pFOPGlow ratios decreased with increasing cell density (Figure 3C). Thus, cell density seemed to be an important determinant of WNT/β-catenin signaling constituting one possible explanation for published inconsistencies.

In summary, the results showed that maintained expression of the internally truncated LRP5 receptor in MCF7 cells appeared necessary for accumulation of transcriptionally active β-catenin.

### WNT3 ligand and LRP5Δ activate transcription synergistically in a DKK1-insensitive manner

**Figure 4:**
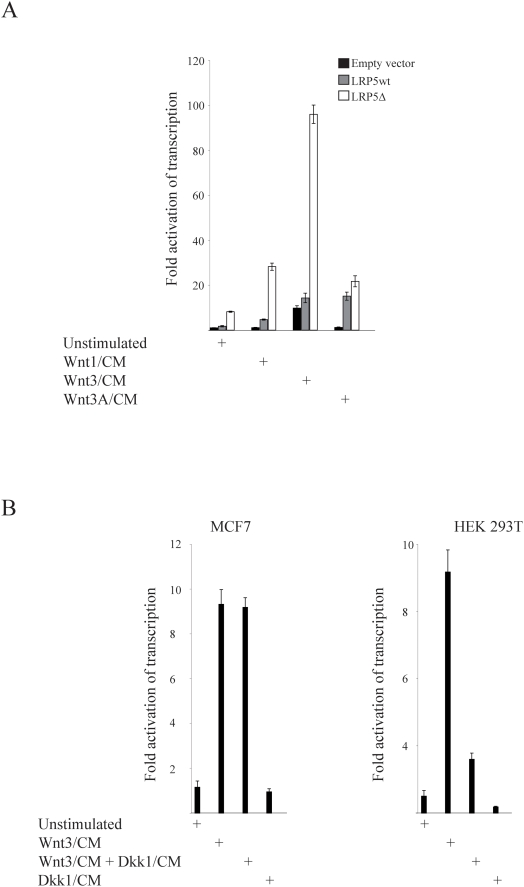
WNT3 ligand and LRP5Δ activate transcription in a DKK1 insensitive manner. (A) MCF7 cells cotransfected with TOPFLASH, LRP5wt or LRP5Δ666–809 expression vectors, CMV-LacZ reference plasmid, followed by incubation in WNT1, WNT3 or WNT3A conditioned medium (CM). CM was from HEK293T cells transiently transfected with expression vectors for the various WNT and DKK1 ligands [26]. The 8-fold (Figure 2D) endogenous β-catenin activity is set to 1 (unstimulated, empty vector). (B) Cotransfection of TOPFLASH and CMV-LacZ reference plasmid to MCF7 cells (left panel) or HEK293T control cells (right panel). Incubation in WNT3 and DKK1 CM. HEK293T cells do not express the LRP5Δ receptor.

We reported previously that WNT3 ligand conditioned medium (CM), but not WNT1 and WNT3A, further activated endogenous β-catenin driven TOPFLASH reporter transcription in sHPT-1 parathyroid tumor cells. WNT1, WNT3 and WNT3A are expressed in MCF7 cells [25], and their effects were determined by transient cotransfections of the TOPFLASH reporter, expression plasmids for LRP5wt or LRP5Δ, and incubation with WNT CMs (Figure 4A). Only WNT3 CM activated the endogenous β-catenin activity in MCF7 (10-fold). Transfection with LRP5Δ increased the endogenous β-catenin activity by 8-fold, and this was further strongly enhanced by WNT3 (12-fold), to a total of 96-fold activation compared to control transfected and unstimulated cells (1.0). Transfection of LRP5wt and stimulation with WNT3 CM resulted in 8-fold activation. WNT1 and WNT3A CM activated transcription in the presence of cotransfected LRP5wt (2.5 and 9-fold) and LRP5Δ (3.5 and 2.5-fold). Thus, WNT1 activated transcription in the presence of cotransfected LRP5Δ in these breast tumor cells, while only WNT3 activity was observed in parathyroid tumors cells [26]. This may reflect the collection of expressed frizzled receptors or other cofactors in the two cell lines.

The LRP5 antagonist DKK1 requires several amino acid residues included in the LRP5Δ truncation, and indeed the DKK1 ligand could not inhibit endogenous LRP5Δ-induced transcriptional activity in parathyroid tumor cells or in LRP5Δ-transfected HEK 293T cells [26]. Similarily, the presence of DKK1 CM did not inhibit WNT3-induced transcriptional activation in MCF7 cells, while inhibition of WNT3-induced endogenous β-catenin activity was observed as expected in HEK 293T control cells (Figure 4B). Thus, the DKK1 ligand failed to inhibit β-catenin signaling *in vitro*, and this may contribute to the total aberrant signaling level in LRP5Δ-positive breast tumors. DKK1 was found to be expressed in all (n = 16) analyzed breast tumors, as determined by immunohistochemistry (not shown).

The data presented so far provide compelling evidence for a major role of the internally truncated LRP5 receptor in sustained aberrant β-catenin signaling in breast cancer.

### LRP5Δ is required for breast tumor cell growth *in vitro* and *in vivo* in SCID mice

**Figure 5:**
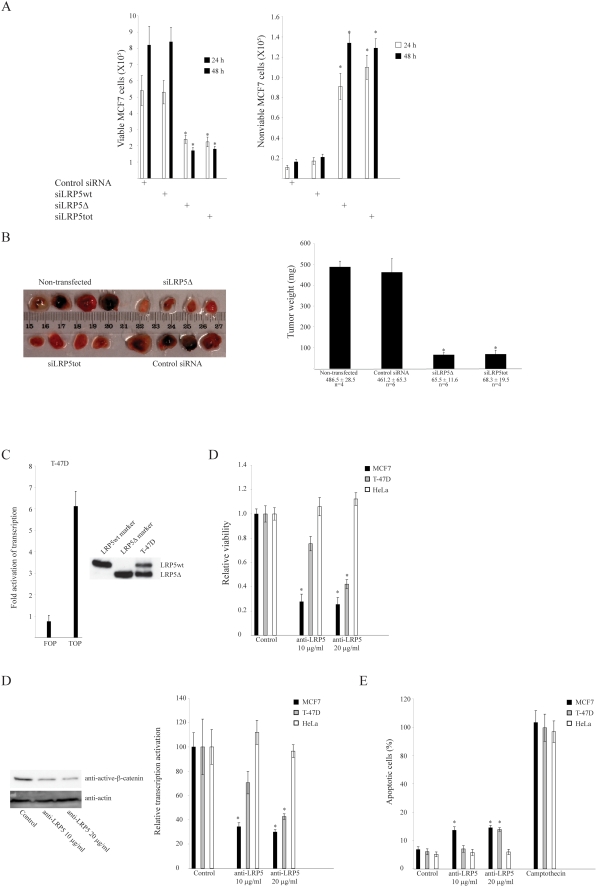
Breast tumor cell growth is dependent on expression of LRP5Δ and continued β-catenin signaling by the receptor. (A) Cell viability after transient siRNA transfection (24 h/48 h). Inhibition of cell growth was not seen in HeLa cells, that were used as control of toxic effects [26]. (B) Breast tumors from SCID mice injected with MCF7 cells pretransfected for 24 h with the indicated siRNAs. The animals were monitored every day and sacrificed after 5 weeks. The tumors were excised and weighed. (C) Endogenous β-catenin activity (left panel) and expression of LRP5wt and LRP5Δ as detected by immunoprecipitation and Western blot analysis (right panel) in T-47D breast cancer cells. (D) Effects on MCF7, T-47D and HeLa cell viability (96 h), non-phosphorylated active β-catenin level (MCF7), and transcription activation (24 h) by β-catenin (TOPFLASH) in the presence of the anti-LRP5 goat polyclonal antibody that recognizes both LRP5wt and LRP5Δ (Figure 1B, Figure 5C). Incubation without antibody or with normal goat serum showed similar effects (Control). (E) Detection of apoptosis during anti-LRP5 antibody incubation (96 h). Camptothecin treatment was used as positive control.

Control siRNA, siLRP5wt, siLRP5Δ, and siLRP5tot were transfected to MCF7 cells and the cell viability was determined 24 hrs and 48 hrs later. Growth inhibition and induction of cell death were seen only with siLRP5Δ, and siLRP5tot at both time points (Figure 5A). Reduced expression of LRP5Δ also lead to growth inhibition and cell death in sHPT-1 parathyroid tumor cells, but not in HeLa cells that do express LRP5wt and not LRP5Δ [26].

Tumor growth was then evaluated in a xenograft SCID mouse model. Tumor growth was significantly reduced in transplants of MCF7 cells pretransfected with siLRP5Δ and siLRP5tot, but not with control siRNA when compared to non-transfected cells (Figure 5B). Thus, LRP5Δ appeared to be necessary for breast tumor cell growth both in cell culture and in SCID mice.

If breast tumor cell growth is dependent on LRP5Δ, as strongly suggested by the above experiments, an appropriate anti-LRP5 antibody may reduce cell viability as well as β-catenin activity. The anti-LRP5 goat polyclonal antibody, that immunoprecipitated both LRP5wt and LRP5Δ (Figure 1B, Figure 2B, and Figure 5C), significantly attenuated the non-phosphorylated active β-catenin level and the β-catenin activity in MCF7 cells, and caused reduced cell viability (Figure 5D). This was not observed in HeLa cells that only express LRP5wt (Figure 5D). We also determined effects of the LRP5 antibody on T-47D breast cancer cells, since these cells expressed both LRP5wt and LRP5Δ, showed nuclear accumulation of β-catenin [25], and displayed endogenous β-catenin activity as determined by the TOPFLASH assay (Figure 5C) [14]. The LRP5 antibody significantly inhibited cell growth and attenuated β-catenin activity also in these cells (Figure 5D). Treatment with the anti-LRP5 antibody induced significant apoptosis in both breast cancer cell lines, and not in HeLa cells (Figure 5E).

Thus, breast tumor cell growth was dependent on maintained expression of LRP5Δ and continued β-catenin signaling by the receptor. The latter result is in line with the observation that WNT antagonist SFRPs were shown to suppress MCF7 and T-47D cell colony formation [25]. Compared to LRP5wt, LRP5Δ activated β-catenin driven transcription more strongly in the presence of WNT3 ligand and in a DKK1-insensitive way, both likely contributing to the oncogenic potential of LRP5Δ. In addition to dephosphorylation/phosphorylation, specific ubiquitination by Rad6B has been suggested to control β-catenin stabilization in MDA-MB-231 breast cancer cells [35]. LRP5Δ was found not to be expressed in this cell line (not shown).

Although the mechanism by which the LRP5Δ receptor is made and how β-catenin signaling is activated remain to be understood, the results presented here strongly support an important role of the truncated LRP5 receptor in mammary gland tumorigenesis, and its expression may present an early event during disease progression. Our results furthermore suggest that antibody therapy directed against LRP5Δ, possibly in combination with chemotherapy [39]–[41], present future treatment options of breast cancer.

## Materials and Methods

### Tissue specimens

Cryosections of nineteen breast cancer specimens, including seventeen invasive ductal carcinoma and two invasive lobular carcinoma [32] were analyzed. Four apparently normal breast tissue specimens from patients with breast cancer were also included in the analyses. Written informed consent was obtained from the patients and approval was obtained from the local ethical committee, Uppsala. cDNA panels containing 96 breast samples covering eight (0, I, IIA, IIB, IIIA, IIIB, IIIC, IV) cancer stages (American Joint Committee on Cancer) were screened for LRP5wt and LRP5Δ (TissueScan Breast Cancer Tissue qPCR arrays BCRT101 and BCRT102, OriGene Technologies, INC. Rockville, MD, USA).

### Detection of LRP5Δ and DNA sequencing

DNA-free total RNA was prepared from breast tissue cryosections and from MCF7 or T-47D cells, and analyzed by RT-PCR using primary and nested primers spanning positions 1992–2932 of LRP5 mRNA as described [26]. RT-PCR analysis of the two breast tissue cDNA panels was performed using the same primers as above, and in addition the nested PCR was also done using a novel forward primer specific for the Δ666–809 truncation: forward primer (position 2012), 5′-TCCACAGGATCTCCCTCGAGACCAATAACAACGACC-3′; and reverse (position 2543), 5′-TTGGACGACTCGATCATGTTGGTGTCCAGGTCGGTC-3′ (GenBank accession number AF064548; http://www.ncbi.nlm.nih.gov/Genbank). The underlined C residue in the forward primer is unique to the Δ666–809 truncation point [26]. The PCR resulted in a 105 base pair fragment. The following conditions were used for the LRP5Δ-specific PCR: The nested PCR amplification was performed with 5 ul primary PCR product, 10 pmol of each primer, 0.2 mM dNTPs, 1× PCR buffer, 1,5 mM MgCl_2_ and 0.25 U Platinum Taq DNA polymerase (Invitrogen Corporation, Carlsbad, California, USA). Denaturation at 95°C for 60 s, followed by 40 cycles of denaturation for 10 s, annealing at 60°C for 20 s and extension at 72°C for 10 s and a final extension at 72°C for 7 min. The LRP5Δ-specific PCR is more sensitive since no competition between LRP5wt and LRP5Δ fragments occurs during the PCR. Additional positive tumors of the cDNA panel was detected by this protocol. A total of sixty-two LRP5 truncated fragments were directly sequenced on ABI 373A using the ABI Prism Dye Terminator Cycle Sequencing Ready Reaction kit (Applied Biosystems, Foster City, California, USA), and all contained the same in-frame deletion (Δ666–809).

### Cell growth determination and apoptosis

MCF7 cells (2×10^5^) were distributed onto 35-mm dishes in DMEM/10% fetal bovine serum and harvested at 24 and 48 hours after siRNA transfection. The number of viable and nonviable cells were determined by using the NucleoCounter (ChemoMetec A/S, Allerod, Denmark) or by using 96-well microplates (10^4^ cells) and the cell proliferation reagent WST-1 (Roche Diagnostics GmbH, Mannheim, Germany). Cells were incubated for 96 hours without serum, with normal goat serum (DakoCytomation, Glostrup, Denmark, # X0907) or with the anti-LRP5 goat polyclonal antibody (Santa Cruz Biotechnology INC., Santa Cruz, CA, # sc-21390). Quantification of cytoplasmic histone-associated-DNA-fragments (mono- and oligonucleosomes) was done using the Cell Death Detection ELISA PLUS kit (Roche Diagnostics). Camptothecin at 0.1 µg/ml was used as positive control of apoptosis.

### Transfection experiments, Western blotting and immunoprecipitation

MCF7 breast tumor cells were transfected with siRNA at least in triplicates at 2×10^5^ cells/35 mm dish with jetSI-ENDO according to the manufacturers recommendations (Poly-Plus-Transfection SAS, Illkirch, France). The various siRNAs have been described previously [26]. MCF7 cells were transfected with a transfection efficiency of approximately 90% (data not shown). Conditioned medium was produced in transiently transfected HEK293T cells, and TOPFLASH/FOPFLASH (Upstate, Lake Placid, USA), pTOPGlow/pFOPGlow [34] TCF luciferase reporter genes or DKK1 promoter luciferase constructs WT short and TBE3,4mut short [38] were transfected to MCF7 cells as described for sHPT-1 cells [26]. The CMV-LacZ reference plasmid was cotransfected and luciferase activities were normalized to β-galactosidase activities. We emphasize that the conditioned medium of WNT1, WNT3, WNT3A, and DKK1 used here might contain uncharacterized signaling molecules induced by the WNTs or DKK1. MCF7, T-47D, and HeLa cells were transfected in 96-well microplates (10^4^ cells) with 0.15 µg TOPFLASH, 0.1 µg CMV-LacZ using 0.5 µl jetPEI (Poly-Plus-Transfection SAS). After 24 h, the cells were incubated further for 24 h without serum, with normal goat serum (DakoCytomation, # X0907) or the anti-LRP5 goat polyclonal antibody (Santa Cruz Biotechnology INC., # sc-21390). Western blotting and immunoprecipitation analyses were done as described [26] using the anti-active-β-catenin [42] mouse monoclonal antibody (Upstate, Lake Placid, USA, # 05-665) and the anti-LRP5 goat polyclonal antibody (Santa Cruz Biotechnology INC., # sc-21390).

### Quantitative PCR analysis of LRP5tot and DKK1 mRNA

cDNA from siRNA transfected MCF7 cells was prepared as described above. The following mRNA-specific PCR primers and labeled probe (5′FAM-sequence-3′TAMRA) were used for quantitative real-time RT-PCR analysis. For LRP5tot: forward, 5′-ATCGACTGTATCCCC GGGGC-3′; reverse, 5′-CACCACGCGCTGGCACACAA-3′; and probe, 5′-CGGACTGTG ACGCCATCTGCC TGC-3′. For 28S rRNA, the Ribosomal RNA Control Reagents (VIC probe) was used (Applied Biosystems, Foster City, California, USA). The following PCR primers were used for DKK1: forward, 5′-TTCTCCCTCTTGAGTCCTTCTG-3′; and reverse, 5′-AGGAGTTCACTGCATTTGGAT-3′. PCR reactions were performed on MyiQ Single-Color Real-Time PCR Detection System (Bio-Rad Laboratories, Inc., Hercules, California, USA) using the TaqMan PCR core Reagent Kit (Applied Biosystems) or the iQ SYBR Green Supermix (Bio-Rad Laboratories, Inc.). Each cDNA sample was analyzed in triplicate. Standard curves for the expressed genes were established by amplifying a purified PCR fragment covering the sites for probes and primers.

### Mouse xenograft model

Two to three week old female Fox Chase severe combined immunodeficient mice (SCID) were used (Taconic, Denmark). The mice were anesthetized with isoflurane (Forene; Abbott, Abbott Park, IL, USA) during the manipulations. Both flanks of each animal were injected subcutaneously (total 200 µl) with MCF7 cells together (1∶1) with BD Matrigel Matrix (BD Biosciences Clontech, Palo Alto, California, USA), after transfection of 10^6^ cells for 24 hours. The animals were monitored every day and sacrificed after 5 weeks. The animal experiments was approved by the Uppsala University board of animal experimentation and was performed according to the United Kingdom Coordinating Committee on Cancer Research guidelines for the welfare of animals in experimental neoplasia [43].

### Statistical analysis

Unpaired *t* test was used for all statistical analyses. Values are presented as arithmetrical mean±SEM. A p value of <0.05 was considered significant.
